# Ureteral obstruction and urinary fistula due to fibrin glue after partial nephrectomy: A case report and review of the literature

**DOI:** 10.3892/ol.2013.1114

**Published:** 2013-01-07

**Authors:** CHAO-JUN WANG, DE-BO KONG, BAI-HUA SHEN, SHUO WANG, BAI-YE JIN, LI-PING XIE, ZHAO-DIAN CHEN

**Affiliations:** Department of Urology, The First Affiliated Hospital, School of Medicine, Zhejiang University, Hangzhou, Zhejiang 310003, P.R. China

**Keywords:** partial nephrectomy, ureteral obstruction, urinary fistula, urinary leakage, fibrin glue

## Abstract

In the present study, we report the case of a 69-year-old female who developed urinary leakage following partial nephrectomy (PN) to remove left renal masses. The results of CT and MR urography revealed left proximal ureteral obstruction and urinary fistula. Reoperation was performed on the 16th postoperative day to explore the left kidney and ureter in order to relieve the obstruction. The left proximal ureter was found to be enfolded by fibrin glue and showed marked stiffness and adhesion during the reoperation. The lesion of the ureter was resected and the ureter was anastomosed with the routine double-J stent. Pathological examination of surgical specimens revealed fat fibrous scar tissue hyperplasia with inflammatory cell infiltration. The patient recovered completely without exudate. Our experience suggests that care should be taken to avoid touching the ureter with fibrin glue during PN surgery.

## Introduction

Partial nephrectomy (PN) is emerging as a nephron-sparing surgery (NSS) in the management of select renal tumours, including benign renal tumours, renal tumours in an anatomically or functionally solitary kidney, renal tumours with potential renal function of the opposite kidney and small renal tumours (T1a and T1b) when the contralateral kidney is normal (1). PN is a technically more complex surgery than radical nephrectomy (RN) and has been shown to be associated with a slightly higher rate of complications (2–4). Ureteral obstruction as a result of surgery is rare and most cases are transient.

The study was approved by the ethics committee of the First Affiliated Hospital, School of Medicine, Zhejiang University, Zhejiang, China. Informed consent was obtained from the patient.

## Case report

**Figure 1:**
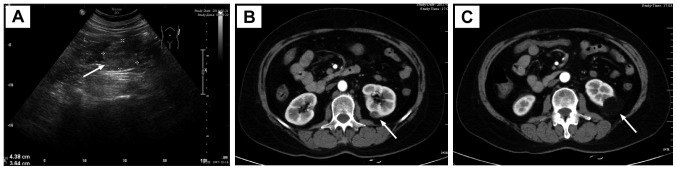
Preoperative imaging showing two renal masses in the lower pole of the left kidney. (A) Renal masses (arrow) were detected by ultrasound. (B and C) Renal masses (arrow) were detected by CT.

A 64-year-old female who had undergone PN in 2011 in The First Affiliated Hospital, Zhejiang University, Zhejiang, China for treatment of a tumour in the left kidney was admitted in February 2011 after 20 days of moderate left lower back pain. Imaging revealed two renal masses in the lower pole of the left kidney (Fig. 1). Following appropriate preoperative preparation, open PN was performed and the two masses were resected. The collection system was not opened during the surgery and the wounds were carefully closed with absorbable sutures. The wounds of the kidney were covered with perirenal fat and fibrin glue was used to adhere the fat to the kidney. The wounds were carefully sutured and a temporary drainage tube was placed near the left kidney. Pathological examination of surgical specimens revealed renal angiomyolipoma. The drainage tube was removed on the 5th postoperative day. The patient recovered completely and was discharged on the 7th postoperative day.

**Figure 2:**
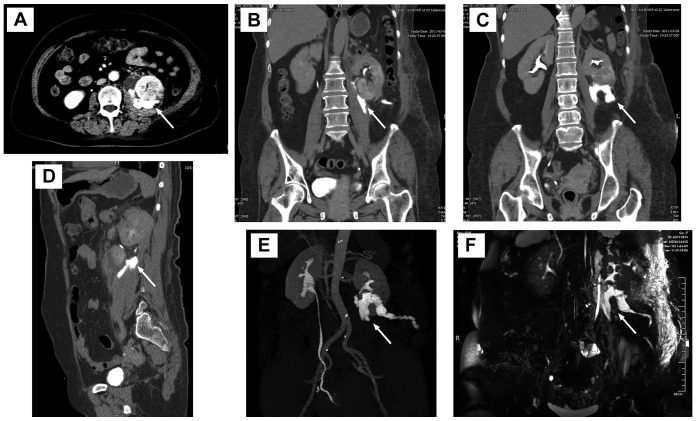
Postoperative imaging showing urinary fistula. (A–E) Urinary fistula (arrow) was detected by CT urography. (F) Urinary fistula (arrow) was detected by MR urography.

**Figure 3:**
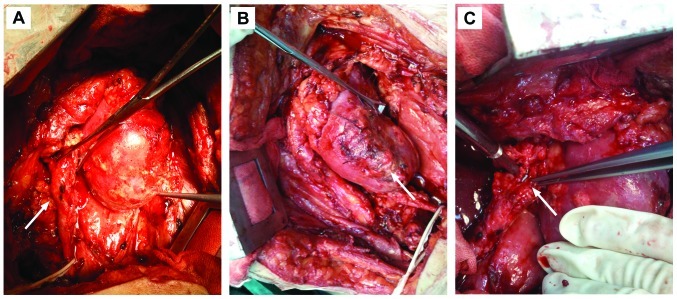
Reoperation was performed to explore the left kidney and ureter. (A) Almost complete occlusion of ureteral stricture was found in the upper ureter (arrow). (B) Surgical wounds of the left kidney (arrow) healed well. (C) The ureter was anastomosed end to end with the routine double-J stent (arrow).

**Figure 4:**
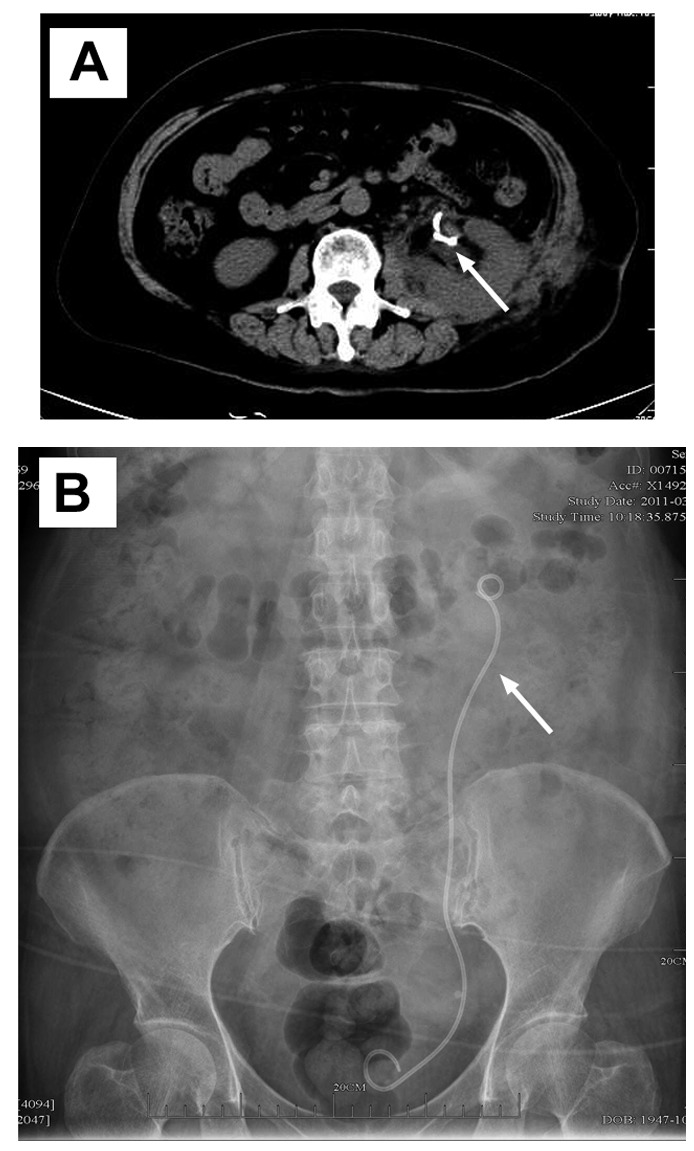
Postoperative (A) CT and (B) plain abdominal X-ray showing the double-J tube (arrow) in the correct position.

However, the patient was re-admitted on the 14th postoperative day due to the discharge of increasingly milky, thin, odourless fluid every day from the unclosed external orifice of the drainage tube. The amount of fluid reached 800 ml on the second day after re-admission. The creatinine concentrations of the fluid and urine were 1847 and 4640 *μ*mol/l, respectively. The result of qualitative analysis of chyle was negative. CT urography (Fig. 2A–E) revealed a clear renal collecting system and no significant hydronephrosis. However, contrast remained in the left kidney and upper ureter and leakage in the perirenal space and along the psoas muscle, and was drained out of the body through the drainage tube. Moreover, no contrast agent was observed in the middle and lower ureter. In MR urography images (Fig. 2F), there was fluid signal in the perirenal space, particularly inside and below the left kidney. The results of CT and MR urography revealed left urinary fistula. A 5-F ureteral catheter was inserted into the left ureter for retrograde pyelography and elevated ∼20 cm but did not pass to the left renal pelvis. Then reoperation was performed on the 16th postoperative day to explore the left kidney and ureter in order to relieve the obstruction, allow unobstructed drainage and save renal function (Fig. 3). During the reoperation, almost complete occlusion of ureteral stricture was found in the upper ureter 5 cm from the left ureteropelvic junction. The ureter near the stricture was enfolded by fibrin glue and exhibited marked stiffness and adhesion. The lesion of the ureter was resected and the ureter was anastomosed end to end with the routine double-J stent (Fig. 4). Pathological examination of surgical specimens revealed fat fibrous scar tissue hyperplasia with inflammatory cell infiltration. The double-J tube was removed by cystoscopy two months after the second surgery. The patient recovered completely without exudate.

## Discussion

PN was first reported in 1884 by Wells for removing two solid circum renal tumours (5). In 1887, Czerny (6) employed the first PN for renal cell cancer (RCC) in a patient with a solitary kidney. Since then, PN has been performed to manage numerous urological diseases, including kidney injuries, renal tumours, renal tuberculosis and partial hydronephrosis. However, its application was limited for postoperative complications until Vermooten (7) demonstrated that PN was oncologically safe for early tumours confined to the kidney and suggested that it should also be used in patients with a healthy contralateral kidney in 1950. Since the early 1990s, PN has been performed mainly as an alternative to RN for small renal tumours (8–11). With the improvement of laparoscopic techniques, laparoscopic RN was gradually established and has become an alternative to open surgery over the last 10 years (12–14). At present, PN is becoming a standard therapy for renal tumours <4 cm in size in a number of centres and is an important therapy for larger T1 tumours (15).

Standard indications for PN are divided into the following categories: absolute, relative and elective indications. PN is absolutely indicated for patients with an anatomically or functionally solitary kidney due to unilateral renal agenesis, previous contralateral nephrectomy or irreversible impairment of contralateral renal function for a benign disorder (16). Relative indications for PN include medical conditions that may impair renal function in the future, such as renal calculus disease, chronic and recurrent pyelonephritis, vesicoureteral reflux disease, renal artery stenosis, hypertension and diabetes mellitus. PN is electively indicated for patients with localised unilateral renal tumours and a healthy contralateral kidney.

It is generally acknowledged that PN has a higher overall complication rate than RN (17). The complications of PN are classified as intraoperative or postoperative. According to previously established criteria (18), intraoperative complications include massive haemorrhage, significant injury to an adjacent organ such as a major vessel, the liver, ureter or pleura, conversion to RN and renal loss. Postoperative complications of PN are classified as urological (such as haemorrhage, urinary leakage/fistula, infection, renal dysfunction, vascular fistula or malformation, positive margin, local recurrence, renal infarct and renal loss) and nonurological (such as cardiac, gastrointestinal, pulmonary, thromboembolic, incisional or other). Postoperative urinary leakage is defined as drainage of >50 ml daily for more than 1 week with fluid biochemistry compatible with urine (19). Urinary leakage persisting for more than 4 weeks after the surgical procedure is generally defined as urinary fistula following PN. Urinary leakage was one of the most common complications of PN. The frequency of urinary leakage occurrence varies between 0.7 and 17.4% in PN (20). Polascik *et al* found that urinary leakage occurred in 21.4% (6 of 28) cases of PN in their earlier surgery, but 0% (0 of 39) cases in their later surgery after the employment of routine injection of the collecting system with dilute methylene blue to identify small defects in the anastomosis (11). Lowrance *et al* considered that urinary leakage was the most notable difference between the PN and RN complications (17). Stephenson *et al* comprehensively reviewed complications of PN and RN in a cohort of 1,049 patients treated at a single centre between 1995 and 2002 (3). In total, 66% of patients in the cohort underwent RN and the remaining 34% underwent PN. The complication rates of urinary fistula were 5.5% for PN and 60.6% (20/33) for all complications associated with PN.

Postoperative urinary leakage occurs due to untight closure of the renal collecting system, ureteral injury or obstruction. Regardless of the cause, any duration of postoperative urinary leakage must be appropriately managed as early as possible. Nonoperative treatment of effective drainage, prevention of infection and avoidance of ureteral obstruction generally resolve the urinary leakage. However, a precondition of nonoperative treatment is no ureteral obstruction. If ureteral obstruction already exists, double-J stenting is required to reasonably save ipsilateral renal function. In the present case, there was discharge of 800 ml of milky, thin, odourless fluid every day from the unclosed external orifice of a drainage tube at 2 weeks after the first surgery. The creatinine concentration of the fluid was almost half of that of urine, but significantly higher than that of serum. The fluid was milky and negative for qualitative analysis of chyle. The results of imaging such as CT and MR urography revealed left urinary fistula. The results of retrograde pyelography made clear that there was proximal ureteral obstruction. Under the circumstances, we considered there was ureteral obstruction and urinary fistula in the left proximal ureter.

Van Poppel *et al* compared the complications of PN and RN and found that reoperations for complications were necessary in 4.4% of the PN group (4). It is generally believed that urinary leakage rarely requires repeat surgery, but in cases of persistent ureteral obstruction and failed ureteral catheterisation, a reoperation may be necessary to relieve obstruction. In most cases, reoperation for urinary leakage results in renal loss. Ramani *et al*(21) performed 200 laparoscopic PNs and only 9 (4.5%) patients had urinary leakage, none of whom required reoperation. Six (3%) required double-J stenting and 2 (1%) required double-J stenting with CT-guided drainage. Meeks *et al*(22) retrospectively reviewed 127 consecutive patients who underwent PN and found that 21 patients (16.5%) experienced a urinary leakage following PN. Most urinary leakages were resolved with prolonged drainage and no surgical reexplorations were required, but 8 patients required a ureteric stent and 2 patients needed percutaneous nephrostomy. In the present case, urinary leakage lasted 2 weeks. Based on imaging findings we considered there was ureteral obstruction and urinary fistula in the left proximal ureter. We performed the reoperation on the 16th postoperative day to explore the left kidney and ureter. The left proximal ureter was found to be enfolded by fibrin glue which was used to adhere the fat and kidney wound in the first surgery. Following surgical reconstruction, the patient recovered completely.

Fibrin glue is a mixture of concentrated autologous fibrinogen and bovine-derived thrombin, which is used to achieve haemostasis during PN (23). Fibrin glue is known for its ability to stop venous oozing from the cut surface of renal parenchymal tissue. It also facilitates haemostatic control as it reproduces the final steps of the coagulation cascade (24). Since haemorrhage is one of the most common complications of PN, fibrin glue is commonly used in open (25,26) and laparoscopic PN (27–29) and fibrin glue-associated complications have not been reported. However, we report unexpected ureteral obstruction and urinary fistula due to fibrin glue. Therefore, we suggest that care must be taken to ensure that the ureter is not in contact with the fibrin glue during PN surgery.

In conclusion, we report fibrin glue-associated ureteral obstruction and urinary fistula following PN. Fibrin glue is commonly used in PN and fibrin glue-associated complication is rare. Therefore, we suggest that the surgeon should take precautions to avoid contact between the ureter with fibrin glue during surgery.

## Abbreviations:

PN: partial nephrectomy;
NSS: nephron-sparing surgery;
RN: radical nephrectomy
